# The complete chloroplast genome of *Indigofera argentea* from Saudi Arabia: comparative genomic analysis within the genus *Indigofera*

**DOI:** 10.3389/fpls.2026.1917737

**Published:** 2026-09-16

**Authors:** Rana M. Alshegaihi

**Affiliations:** Department of Biological Sciences, College of Science, University of Jeddah, Jeddah, Saudi Arabia

**Keywords:** chloroplast genomes, comparative plastomics, cpSSR, *Indigofera*, NGS, phylogenetics

## Abstract

**Introduction:**

The genus *Indigofera* is broadly distributed across tropical and subtropical regions and displays high morphological diversity, yet its phylogenetic relationships remain poorly resolved. This study aimed to characterize the complete chloroplast genome of *Indigofera* argentea from Saudi Arabia and evaluate its comparative genomic and phylogenetic relationships with other *Indigofera* species.

**Methods:**

The complete chloroplast genome of *I. argentea* was characterized and compared with seven publicly available *Indigofera* plastomes. Comparative analyses included genome structure, gene content and organization, GC content, IR boundary variation, codon usage, simple sequence repeats (SSRs), nucleotide diversity, single nucleotide polymorphisms (SNPs), selection pressure, and phylogenetic relationships.

**Results:**

The *I. argentea* chloroplast genome is 158,639 bp, exhibiting the typical quadripartite structure and encoding 128 genes (83 protein-coding, 37 tRNA, and 8 rRNA genes), with an overall GC content of 35.9%. Comparative analyses revealed strong structural conservation among *Indigofera* plastomes in gene content, gene order, GC content, and IR boundary organization, although minor variations were detected at the LSC, IR, and SSC junctions, which did not affect overall genome structure. Codon usage showed a bias toward A/U-ending codons, consistent with the AT-rich genome composition, and RSCU values varied among genomic regions. Repeat analysis identified 725 SSRs, predominantly located in the LSC region. Four mutation hotspot regions, *trn*K-*rbc*L, *trn*L-*rps*4, *ndh*F-*trn*L, and *ycf*1, exhibited elevated nucleotide diversity, and a total of 2,059 SNPs were detected, largely concentrated at the third codon position, with *ycf*1, *rpo*C2, and *ndh*F showing the highest levels of variation. Most protein-coding genes were under strong purifying selection, although 13 genes exhibited signs of positive selection, with *ycf*1, *rps*18, and *rps*19 showing the strongest signals. Phylogenetic analyses resolved *Indigofera* as a strongly supported monophyletic group, identifying four distinct clades and supporting the use of complete plastomes for resolving relationships within the genus.

**Discussion:**

The conserved plastome structure, together with the identified mutation hotspots, SNPs, and genes showing signals of positive selection, provides valuable insights into plastome evolution and lineage diversification within *Indigofera* and highlights informative genomic regions for future comparative and phylogenetic investigations.

## 1 Introduction

The flora of Saudi Arabia is highly diverse, shaped by the convergence of East African, Mediterranean, and Irano-Turanian phytogeographic elements and the country’s geographic position between Africa and South Asia (Alfarhan, 1999). Ongoing botanical surveys have led to the discovery of several taxa newly recorded for the Saudi flora (Fayed and Al-Zahrani, 2007; Masrahi, 2015). Within this rich floristic diversity, Fabaceae represents one of the largest and most economically important angiosperm families (Christenhusz, 2016). It is widely distributed globally and ranks as the third-largest family of land plants, after Orchidaceae and Asteraceae, comprising approximately 751 genera and nearly 19,000 described species. The family also includes several exceptionally large genera, *Acacia*, *Astragalus*, *Mimosa*, *Crotalaria*, and *Indigofera*, which together account for nearly 25% of all legume species (Kambhar et al., 2017). Given the ecological and economic significance of Fabaceae in Saudi Arabia, understanding the genomic diversity of key genera like *Indigofera* is critical for conservation and utilization.

The genus *Indigofera*, a member of the subfamily Papilionoideae, comprises approximately 700–750 species of herbs, shrubs, and small trees distributed throughout tropical and subtropical regions worldwide, with major diversity centers in Africa and Madagascar, and about 20 species reported in Saudi Arabia, mainly in the western and southwestern regions (Chaudhary and al-Waţanīyah, 2001; Schrire, 2005; Schrire et al., 2009). The genus is taxonomically challenging, as many species exhibit overlapping morphological traits and broad geographic distributions, making species identification and delimitation difficult (Schrire, 1995). Several species are cultivated for natural indigo dye production, forage and soil improvement, ornamental use, and traditional medicine due to their antimicrobial, antiparasitic, and hepatoprotective properties (Prakash et al., 2007; Marquiafável et al., 2009; Renukadevi and Sultana, 2011; Schrire, 2013; Agustarini et al., 2022). *Indigofera argentea* Burm.f is a hardy, silver-pubescent shrub, typically 30–60 cm tall, common to the arid regions of Saudi Arabia, particularly in the southwestern areas. Often found in dry shrublands, this Fabaceae member has 5–7 leaflet leaves and produces pink-red flowers and small, 2–5-seeded pods (Chaudhary, 2000).

With the advances in next-generation sequencing, the acquisition of complete genomes has become increasingly accessible (Levy and Myers, 2016). Chloroplast genomes (cpDNA) are widely used in plant phylogeny and taxonomy due to their conserved structure, uniparental inheritance, and moderate mutation rates (Wicke et al., 2011; Zhao et al., 2019). Typically, cp genomes are circular and quadripartite, consisting of a large single-copy (LSC) region, a small single-copy (SSC) region, and two inverted repeats (IR) (Palmer and Stein, 1986; Jansen et al., 2005; Ruhsam et al., 2015). Complete chloroplast genome sequences further provide important insights into evolutionary history and natural selection and have contributed significantly to understanding evolutionary mechanisms in plants (Shanker and Sharma, 2012). Several studies have characterized complete chloroplast genomes in selected *Indigofera* species. For instance, Zhang et al (Zhang et al., 2022). analyzed *Indigofera stachyodes*, Zhao et al (Zhao et al., 2023). investigated *Indigofera amblyantha* and *Indigofera pseudotinctoria*, and Zhou et al (Zhou et al., 2023). conducted comparative plastome analyses across 17 *Indigofera* species. Despite these advances, plastome data remain unavailable for many members of the genus, especially from the Palaeotropical clade, limiting comprehensive insights into *Indigofera* phylogeny, biogeographic history, and evolutionary diversification.

To address these gaps, we assembled and annotated the complete chloroplast genome of *I. argentea* and conducted comparative analyses with seven other publicly available *Indigofera* plastomes representing Pantropical, East Asian, Tethyan, and Palaeotropical clades (Zhang et al., 2022). Genome structure, tandem repeats, codon usage, and evolutionary patterns, and construct phylogenetic relationships within the genus were examined to provide genomic resources that improve taxonomic resolution and deepen our understanding of *Indigofera* evolution and diversification.

## 2 Materials and methods

### 2.1 Plant material, DNA extraction, and sequencing

Fresh leaf specimens of *I. argentea* (Aldurydah) were harvested from Jeddah, Saudi Arabia (ring road: 21°47’11.9” N, 39°08’30.0” E). Genomic DNA was subsequently extracted from the leaf tissue utilizing the WizPrep™ gDNA Mini Kit (Cell/Tissue; WIZBIO, Seoul, South Korea) following the manufacturer’s instructions. DNA integrity was assessed by 1% agarose gel electrophoresis, while DNA concentration and purity were measured using a Quantus™ Fluorometer (Promega, Madison, WI, USA). For further processing, the extracted gDNA was stored at a constant temperature of −20 °C. Paired-end sequencing libraries were generated from high-quality genomic DNA using the Illumina TruSeq DNA Sample Preparation Kit (Illumina, San Diego, CA, USA), and paired-end sequencing was performed with a 350-bp insert size. Sequencing was performed on an Illumina HiSeq 4000 platform with 150 bp reads (Novogene, UK).

### 2.2 Chloroplast genome assembly, annotation and codon usage bias analysis

Raw sequencing data for the chloroplast genome of *I. argentea* were quality-trimmed, filtered, and assembled using Geneious Prime (Kearse et al., 2012) following a single-contig assembly approach (Magdy et al., 2019). The resulting assembly was annotated with GeSeq (Tillich et al., 2017), using the chloroplast genome of *I. linifolia* (GenBank accession no. NC_047353.1) as a reference. The annotated circular genome maps were generated and visualized with OGDRAW (Greiner et al., 2019). Geneious Prime was used to retrieve basic chloroplast genome features, including sequence length, GC content, lengths of the LSC, SSC, and IR regions, gene number and type, and overall nucleotide composition.

Codon usage bias was assessed in MEGA v12 (Kumar et al., 2024), by calculating relative synonymous codon usage (RSCU) values. Preferred and unpreferred codons were then identified to evaluate codon usage patterns across all protein-coding genes.

### 2.3 Comparative analysis of the *Indigofera* chloroplast genomes

In addition to the newly generated chloroplast genome of *I. argentea*, complete chloroplast (cp) genome sequences of seven *Indigofera* species (*Indigofera carlesii* Craib, *Indigofera linnaei* Ali, *Indigofera amblyantha* Craib, *Indigofera bungeana* Walp, *Indigofera wightii* Graham, *Indigofera hirsuta* L., and *Indigofera linifolia* (L.f.) Retz.) were retrieved from the NCBI database and included in all comparative analyses (**Table 1**). Taxon sampling followed the established four-clade framework of *Indigofera*, with emphasis on species from the same clade as *I. argentea* and its closely related or sister clade to assess its phylogenetic placement (Zhou et al., 2023).

**Table 1:** The general characteristics of the eight *Indigofera* chloroplast genomes.

*Indigofera* sp.	Accession	Size				GC%	Total genes (CDS/tRNA/rRNA)
		Total	LSC	SSC	IR	Total (LSC/SSC/IR)	
*I. argentea*	Aldurayda	158639	88940	19075	50624	35.9% (33.3/29/43)	128 (83/37/8)
*I. carlesii*	OQ147469	159172	89407	19063	50702	35.7% (33.1/28.9/42.9)	
*I. linnaei*	OQ147475	159450	89890	18940	50620	35.7% (33/29.1/42.9)	
*I. amblyantha*	OQ134123	159686	89849	19083	50754	35.6% (32.9/28.9/42.9)	
*I. bungeana*	OQ147468	159777	90093	19072	50612	35.6% (32.9/28.9/42.9)	
*I. wightii*	OQ147480	159787	90109	19098	50580	35.7% (33/28.9/43)	
*I. hirsuta*	OQ147472	159873	90361	18858	50654	35.5% (32.8/28.8/42.9)	
*I. linifolia*	NC_047353	160040	90459	18935	50646	35.8% (33.1/29.4/42.9)	

Simple sequence repeats (SSRs) were detected using Phobos V3.3 (http://www.rub.de/ecoevo/cm/cm_phobos.htm, accessed on 9 February 2026) with default settings for imperfect search mode to capture SSRs with minor sequence variations, with repeat unit lengths ranging from 2 bp to 10 bp, implemented within Geneious Prime. Additionally, long repeat sequences, including direct and palindromic repeats, were identified using the Vmatch software package (van Melle, 1998).

The boundaries and junctions between the LSC, SSC, and IR regions of the chloroplast genomes from eight *Indigofera* species were examined using the online IRscope tool (https://irscope.shinyapps.io/irapp/; accessed 8 February 2026).

To assess genetic diversity and identify hypervariable regions among *Indigofera* species, complete chloroplast genomes from eight species were aligned using MAFFT (Katoh and Standley, 2013), implemented in Geneious Prime. Nucleotide diversity (π) was calculated using DnaSP (Rozas et al., 2017) based on a sliding window analysis with a window length of 600 bp and a step size of 200 bp.

Single-nucleotide polymorphisms (SNPs) in protein-coding sequences of *Indigofera* were identified using Geneious Prime, with *I. argentea* designated as the reference. SNP calling was performed using a minimum coverage of 2 and a minimum variant frequency of 0.25%, and variants were classified as synonymous or nonsynonymous. Based on these classifications, the nonsynonymous to synonymous substitution ratio (dN/dS, ω) was calculated to assess selective pressures acting on protein-coding genes.

### 2.4 Phylogenetic analysis

Multiple sequence alignments of complete chloroplast genomes from eight *Indigofera* species were generated using MAFFT, as implemented in Geneious Prime, and trimmed to a final alignment length of 176,250 bp. Maximum likelihood (ML) analyses were performed using the complete chloroplast genome as well as the LSC, SSC, and IR regions. Phylogenetic trees were inferred with FastTree v2 (Price et al., 2010), under the generalized time-reversible (GTR) nucleotide substitution model using default parameters. Bootstrap support was assessed using the default settings in FastTree v2. The complete chloroplast genome of *Arachis hypogaea* (GenBank accession no. KX257487.1) was set as the outgroup.

## 3 Results

### 3.1 Characteristics of the *Indigofera argentea* chloroplast genome

The newly sequenced chloroplast genome of *I. argentea* is 158,639 bp in length and exhibits the typical quadripartite structure characteristic of angiosperm chloroplast genomes, consisting of an LSC region of 88,940 bp, an SSC region of 19,075 bp, and a pair of IR regions of 25,312 bp each (**Figure 1**; **Table 1**).

**Figure 1:**
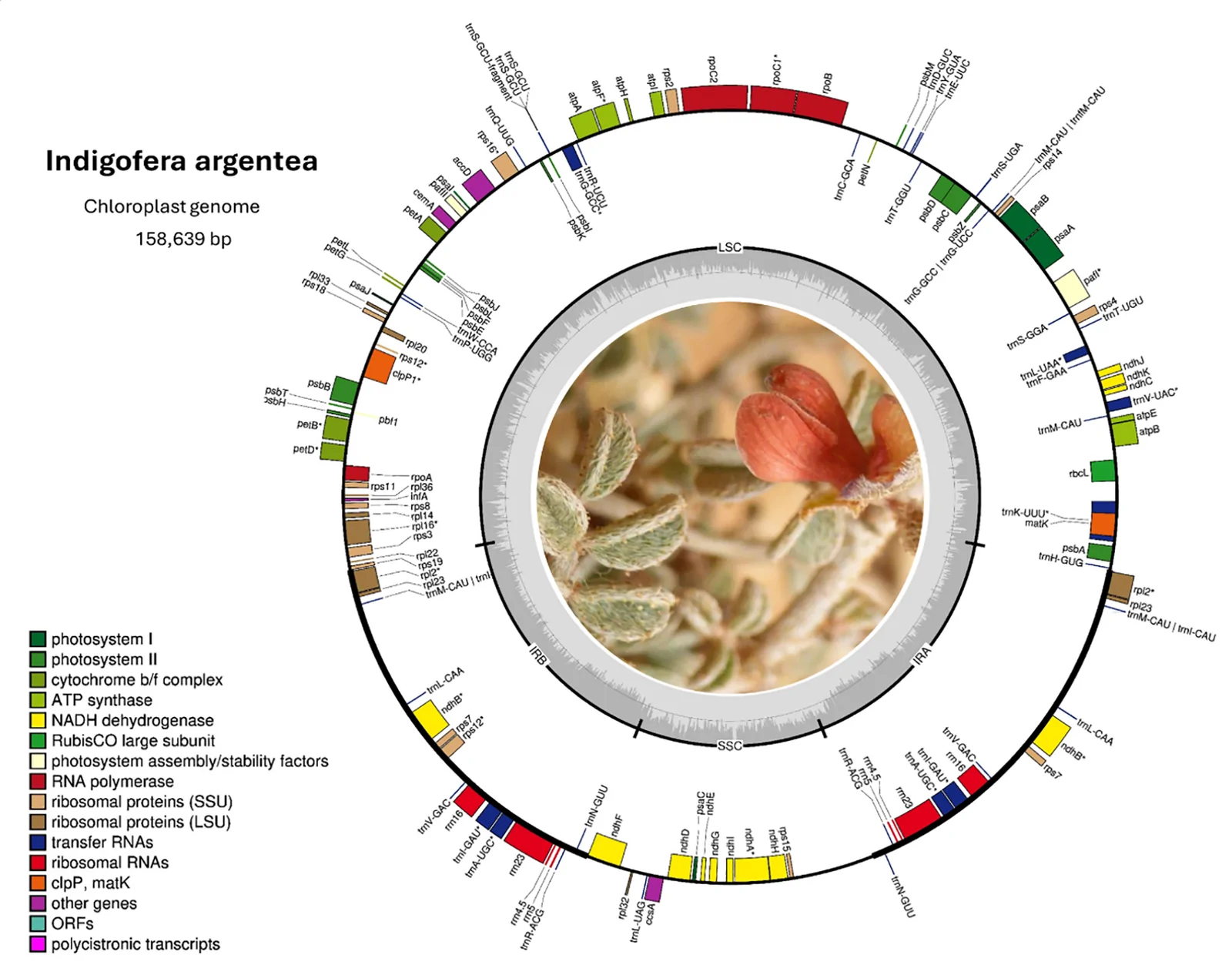
Circular map of the complete chloroplast genome of *I. argentea*. Inside the circle are genes transcribed in the clockwise direction, and genes outside the circle are transcribed in an anticlockwise direction. Genes are color-coded according to functional categories. The innermost circle indicates the LSC, SSC, and IR regions, and the dark gray plot represents GC content across the genome.

The plastome has an overall GC content of 35.9%, exhibiting the typical AT-rich composition (64%) of angiosperm plastomes. GC content varies among regions, being highest in the IR regions (42.6%) and lower in the SSC (33.3%) and LSC (27.3%) regions, consistent with the GC enrichment associated with IR localized rRNA genes. The nucleotide composition is 32.1% A, 32.0% T, 17.8% C, and 18.1% G. Coding regions comprise 55.9% (88,684 bp) of the genome, with CDS genes accounting for the largest proportion (76,829 bp; 48.4%), followed by rRNA (9,062 bp; 5.7%) and tRNA genes (2,793 bp; 1.8%), while IGS constitute 31.8% (50,423 bp) of the plastome.

Comparative analysis of publicly available *Indigofera* chloroplast genomes retrieved from the NCBI database revealed a high degree of structural conservation across the genus (**Table 1**). All genomes exhibited conserved gene order and synteny, with no detectable structural rearrangements. Overall genome organization was highly conserved, with total genome sizes ranging from 159,172 bp in *I. carlesii* to 160,040 bp in *I. linifolia*. The chloroplast genomes of *Indigofera* species exhibited highly similar GC contents, ranging from 35.6% in *I. amblyantha* to 35.8% in *I. linifolia*, reflecting limited compositional divergence within the genus (**Table 1**).

All examined plastomes encoded a total of 128 genes, including 83 coding DNA sequences (CDS), 37 tRNA genes, and eight rRNA genes, with no variation in gene number among species (**Supplementary Table 1**). Of these, 112 genes were present as single copies, whereas 16 genes were duplicated within the IR regions, including five CDS (*rps*7, *ndh*B, *ycf*2, *rpl*23, *rpl*2), seven tRNAs (*trn*N^GUU^, *trn*R^ACG^, *trn*A^UGC^, *trn*I^GAU^, *trn*V^GAC^, *trn*L^CAA^, *trn*I^CAU^), and four rRNAs (rrn5, rrn4.5, rrn23, rrn16). Among the 128 genes, 24 contain introns; clpP and ycf3 harbor two introns each, while the remaining 22 genes possess a single intron, comprising 12 CDS (*rpo*C1, *atp*F, *rps*16, *clp*P1, *pet*B, *pet*D, *rpl*16, *rpl*2, *ndh*B, *rps*12, *ndh*A, *ndh*B) and eight tRNA genes (*trn*K^UUU^, *trn*V^UAC^, *trn*L^UAA^, *trn*G^GCC^, *trn*I^GAU^, *trn*A^UGC^).

### 3.2 Analyses of SSR and long repeat sequence

Repetitive sequence analysis of the *I. argentea* chloroplast genome revealed a total of 1,028 simple sequence repeats (SSRs), which were classified according to repeat unit length, ranging from dinucleotide to 8-nucleotide repeats. Dinucleotide repeats were the most prevalent class, accounting for 458 SSRs (45%), followed by hexanucleotide repeats with 175 occurrences (17%). Analysis of their genomic distribution revealed a clear regional bias, with most repeats concentrated in the LSC region, whereas the IR and SSC regions exhibited comparatively lower densities.

A comparative analysis of SSRs across eight *Indigofera* chloroplast genomes identified a total of 8,426 repeats. The number of SSRs per plastome ranged from 1,028 in *I. argentea* to 1,087 in *I. hirsuta*, indicating a largely conserved SSR abundance among the examined species. Dinucleotide repeats constituted the predominant class, with 455–486 repeats per genome, accounting for approximately 45% of the total SSRs on average. Trinucleotide repeats represented the second most abundant category (149–175 per genome; ~16%), followed by hexanucleotide (~15%), pentanucleotide (~14%), and tetranucleotide repeats (~11%). In contrast, repeats of longer than six nucleotides occurred only in a few species (**Figure 2A**).

**Figure 2:**
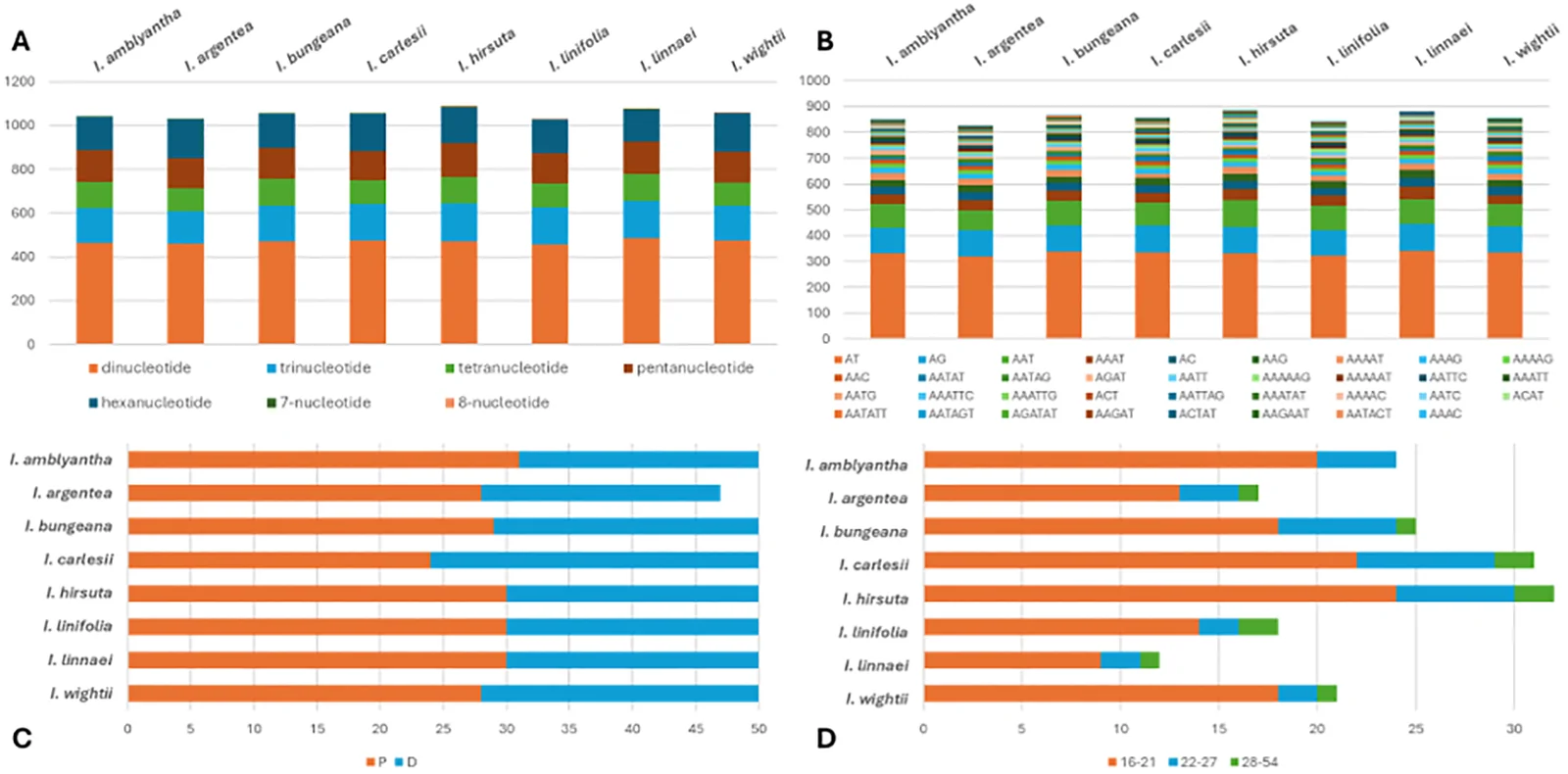
Characterization of SSRs and long repeat sequences in the chloroplast genomes of *Indigofera* species. **(A)** Number of SSRs identified in each repeat class across the analyzed plastomes. **(B)** Abundance of identified SSRs across repeat classes detected in all plastomes, illustrating interspecific variation among Indigofera species. **(C)** Number and types of long repeat sequences detected in each species, including palindromic and direct repeats. **(D)** Length distribution of long repeat sequences across different size categories.

Analysis of repeat composition across all eight species revealed a conserved pattern with interspecific variation and a clear AT-rich bias. Dinucleotide repeats were predominantly composed of AT and AG repeats, whereas trinucleotide repeats were mainly represented by AAT and AAG. Similarly, tetranucleotide repeats were dominated by AAAT and AAAG repeats, while pentanucleotide and hexanucleotide repeats were largely A/T-rich, including repeats such as AAAAT/AAAAG and AAAAAT/AAAAAG, respectively (**Figure 2B**).

In addition to SSRs, two repeat types were detected across the analyzed plastomes: palindromic and direct repeats. Palindromic repeats ranged from 24 in *I. carlesii* to 31 in *I. amblyantha*, whereas direct repeats varied from 19 in *I. argentea* and *I. amblyantha* to 26 in *I. carlesii* (**Figure 2C**). In terms of length distribution, repeats of 10–15 bp were the most abundant (35 - 48), followed by 16–21 bp repeats (9 - 24). Repeats of 22–27 bp were less common (2 - 7), whereas the longest repeats (28–54 bp) were rare, ranging from 0 to 2 across the analyzed plastomes (**Figure 2D**).

### 3.3 Expansion and contraction of IR regions

The IR regions represent the most structurally conserved components of the chloroplast genome; however, small expansions and contractions at their boundaries are recognized as the primary drivers of plastome length variation during evolution. The four principal junctions, JLB (IRb/LSC), JSB (IRb/SSC), JSA (SSC/IRa), and JLA (IRa/LSC), define the transitions between single-copy and IR regions. Comparative assessment of these junctions across the eight *Indigofera* plastomes revealed a highly conserved boundary configuration, with only minor interspecific shifts in gene positioning relative to the junction sites.

Comparative analysis of IR boundary positions among the eight *Indigofera* plastomes revealed only minor expansions and contractions (**Figure 3**). At the LSC/IRb (JLB) boundary, *rps*19 crossed the junction with most of the gene in the LSC (264–277 bp) and a short segment in IRb (2–15 bp), while *rpl*2 was entirely within IRb. At the IRb/SSC (JSB) boundary, *ycf*1 spanned the junction, producing a truncated fragment in IRb (446–477 bp) and a full-length sequence in SSC. At the SSC/IRa (JSA) boundary, *ycf*1 extended into IRa (5,357 - 5,381 bp) with an overlap of 442–478 bp, whereas *ndh*F remained near JSB with minimal shifts (1–12 bp). At the IRa/LSC (JLA) boundary, *rpl*2 was fully in IRa, and *trn*H was in the LSC, 63–75 bp from the junction.

**Figure 3:**
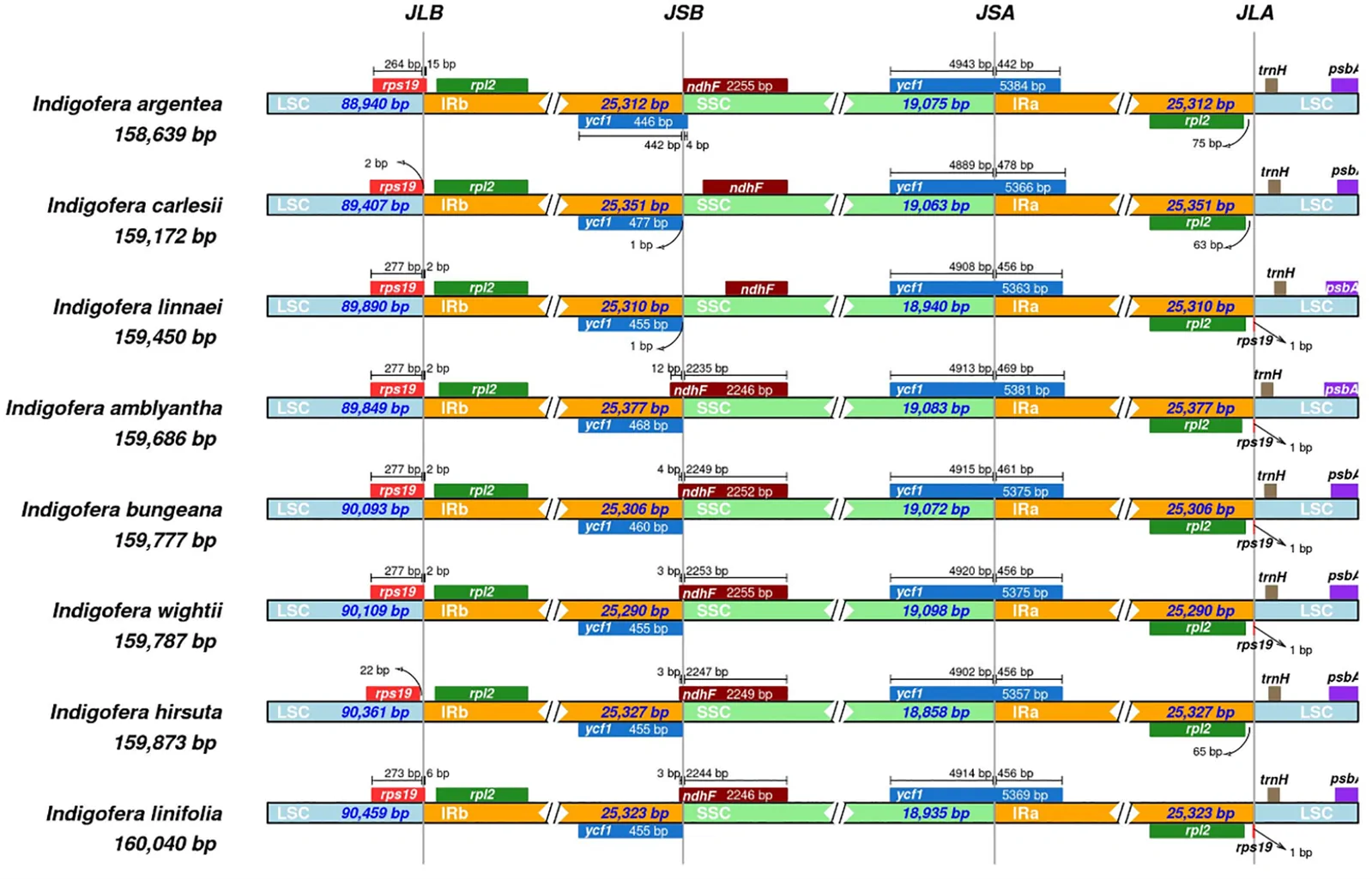
Comparative analysis of the junctions between LSC, SSC, and IR regions across eight *Indigofera* chloroplast genomes. Genes are represented by colored boxes, with arrows indicating their positions and orientation near the junctions. The junction sites are denoted as JLB (IRb/LSC), JSB (IRb/SSC), JSA (SSC/IRa), and JLA (IRa/LSC), illustrating the relative positions and subtle expansions or contractions of genes at the IR boundaries among species.

### 3.4 Codon usage patterns in the chloroplast genomes

A total of 25,611 codons were identified within the CDS of the *I. argentea* chloroplast genome. The RSCU analysis was performed across all 64 codons encoding the 20 standard amino acids. Methionine (AUG) and tryptophan (UGG) showed no codon bias (RSCU = 1), whereas other amino acids displayed varying degrees of preference for synonymous codons. Arginine, leucine, and serine exhibited the highest codon degeneracy, each being encoded by six codons, while most other amino acids were represented by two or four codons. Isoleucine was encoded by three synonymous codons, consistent with the universal genetic code.

Analysis of the RSCU values revealed a clear preference for codons ending with A or U, reflecting the AT-rich composition of the chloroplast genome (**Figure 4**). For example, Leu favored UUA and UUG, Ile preferred AUU, and other amino acids, including Val, Pro, Thr, Ala, Tyr, His, Gln, Asn, Lys, Asp, Glu, Cys, Arg, and Gly, also exhibited higher usage of A/U ending codons compared to C/G ending codons. Some codons, such as AGU (Ser) and UGG (Trp), retain unbiased usage, highlighting conserved codon selection for specific amino acids across the genome.

**Figure 4:**
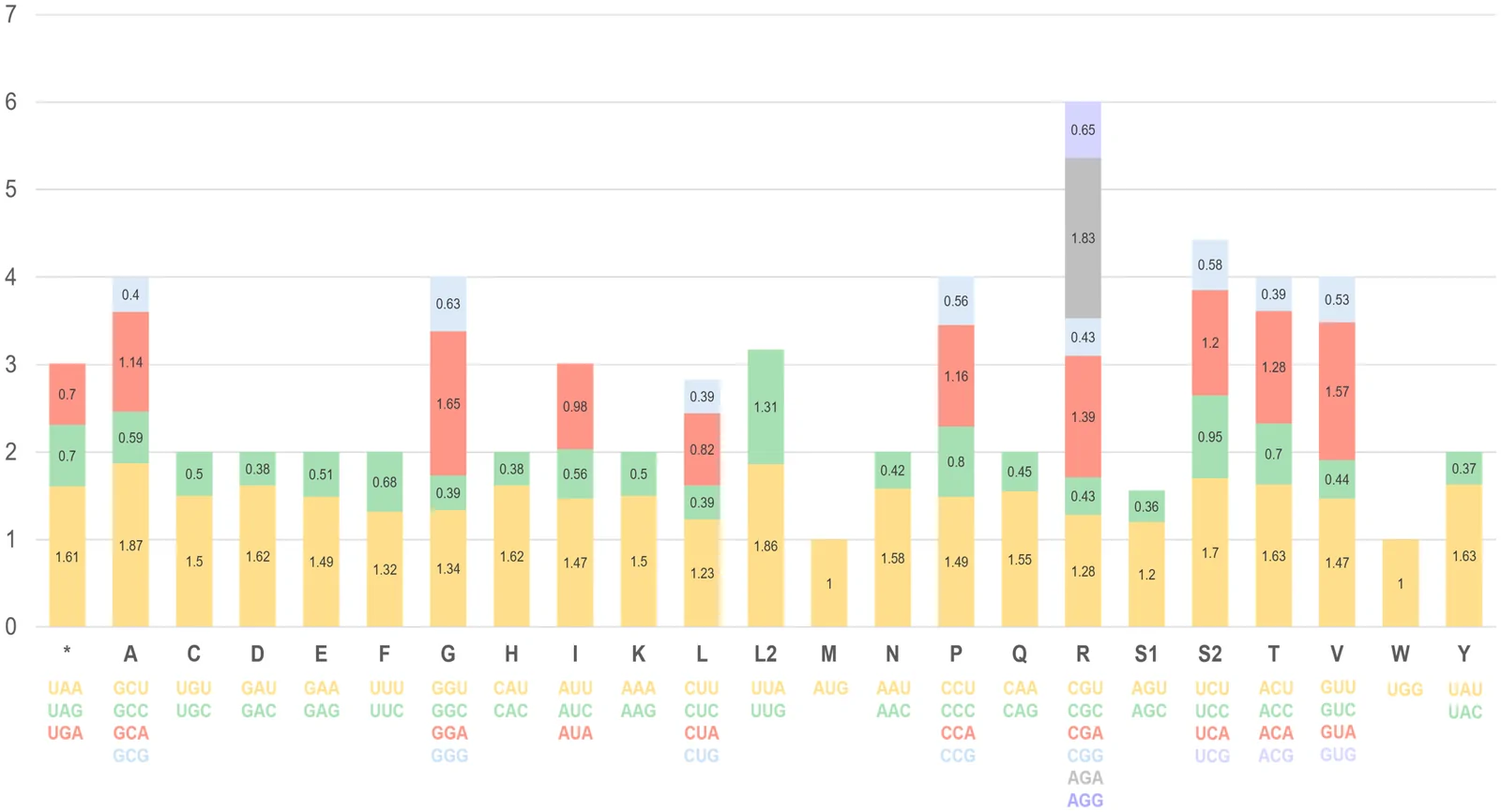
Relative synonymous codon usage (RSCU) values of protein-coding sequences in the *I. argentea* chloroplast genome, highlighting codon usage bias. Different colors distinguish individual codons and amino acids, which are indicated by their single-letter abbreviations.

Among all amino acids, Leu was the most abundant, representing 10.6% of all codons, followed by Ile at 9.0%, whereas Cys was the least frequent, comprising only 1.3% of codons. At the individual codon level, AUU (Ile) was the most frequently used, with 1,119 occurrences, while UGC (Cys) was the rarest, appearing only 95 times throughout the genome.

### 3.5 Divergence hotspot regions

To identify highly variable regions for species discrimination in *Indigofera*, a sliding-window analysis was performed on the aligned chloroplast genomes (**Figure 5A**, **Supplementary Table 2**). Nucleotide diversity (π) across the entire alignment ranged from 0 to 0.05696, with an average of 0.0074. The number of segregating sites per window varied from 0 to 124, with an average of 108.7. Based on the top 10% of π values, four mutation hotspot regions were identified after merging overlapping windows: trnK-*rbc*L, trnL-*rps*4, *ndh*F-trnL, and *ycf*1. These regions exhibited the highest nucleotide diversity, with π values ranging from 0.043 to 0.057, and correspondingly high numbers of segregating sites.

**Figure 5:**
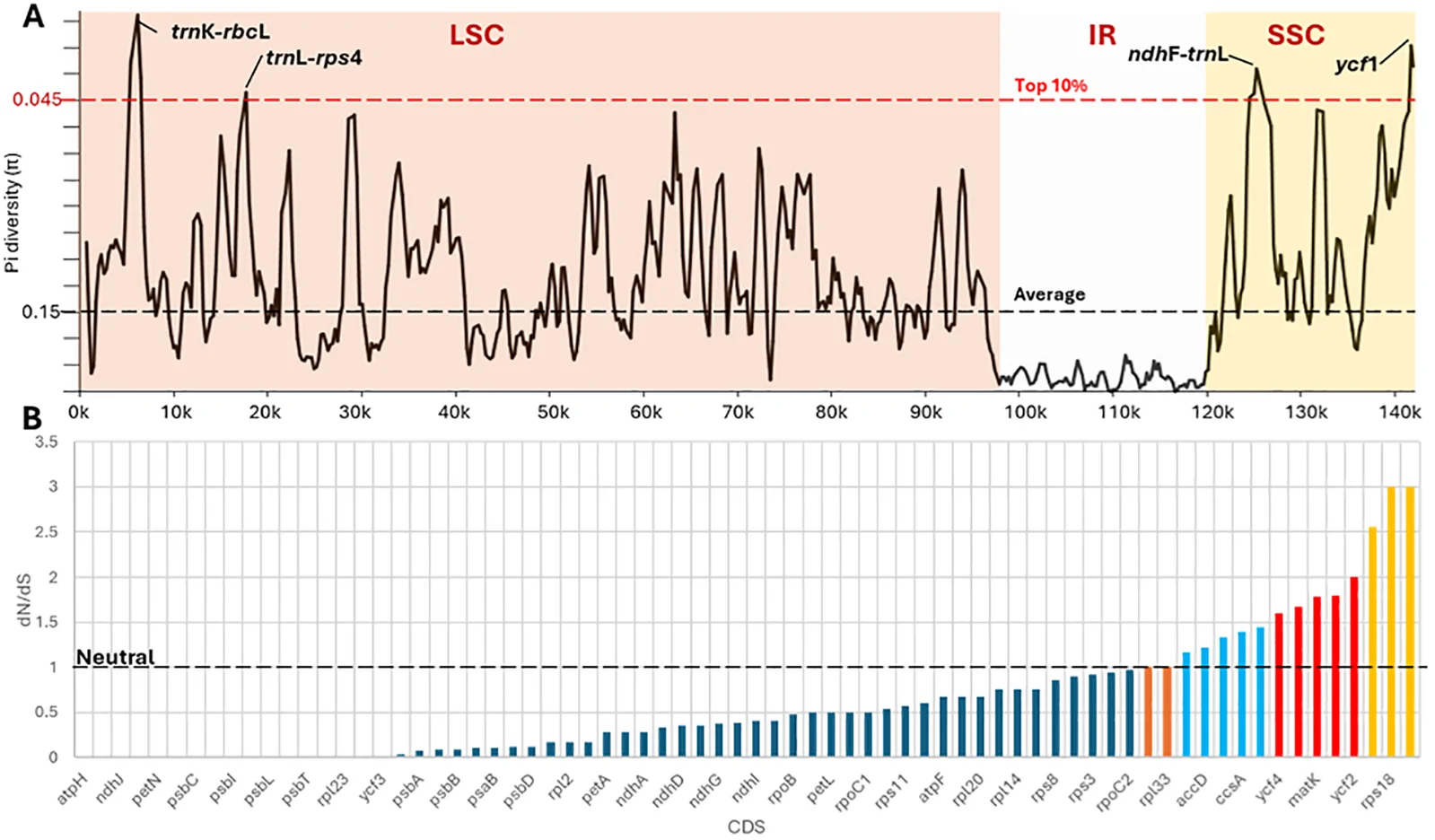
Patterns of nucleotide diversity (π) and selective pressure in Indigofera chloroplast genomes. **(A)** Sliding-window analysis of nucleotide diversity across complete chloroplast genomes of Indigofera species. The highest variation hotspots are annotated on the graph. **(B)** Selective pressure on chloroplast protein-coding genes based on dN/dS (ω) ratios. Genes without bars or with blue bars represent ω < 1 (purifying selection). Two genes (psaI and rpl33), shown in orange, represent neutral evolution (ω = 1). Genes with ω > 1 represent positive selection and are color-coded by magnitude: light blue (ω = 1.1–1.4), red (1.6–2.0), and yellow (2.5–3.0).

Single-nucleotide polymorphisms (SNPs) were identified across *Indigofera* species using *I. argentea* as the reference genome (**Supplementary Table 3**). A total of 2,059 SNPs were detected across CDS regions, including 1,116 transitions and 943 transversions, comprising 1,109 synonymous and 950 non-synonymous substitutions. Analysis of codon position distribution showed that substitutions were unevenly distributed across coding sites, with the majority occurring at the third codon position (1,216 SNPs), followed by the first codon position (490 SNPs) and the second codon position (353 SNPs). At the gene level, substantial variation in SNP distribution was observed across chloroplast protein-coding genes. The hypothetical protein gene *ycf*1 contained the highest number of SNPs (508), followed by the RNA polymerase gene *rpo*C2 (130) and the NADH dehydrogenase gene *ndh*F (123). Other genes with relatively high SNP counts included *ycf*2 (90), *mat*K (89), *rpo*B (74), and *acc*D (62). Moderate levels of polymorphism were detected in several genes, such as *psb*B (39), *atp*A (35), *psb*C (32), *atp*B (32), and *rpo*A (31). In contrast, several genes showed only a few polymorphic sites, including *psa*I, *psa*J, *pet*L, *pet*N, *psb*L, *psb*E, *psb*M, *psb*T, and *rpl*23, each containing between one and three SNPs.

To investigate selective pressures acting on chloroplast protein-coding genes, the ratio of non-synonymous to synonymous substitution rates (dN/dS, ω) was calculated across aligned coding sequences (**Figure 5B**, **Supplementary Table 3**). The majority of genes (59) exhibited ω < 1, indicating strong purifying selection across most chloroplast coding regions. These included photosystem genes (*psaA, psaB, psaC, psaJ, psbA, psbB, psbC, psbD, psbE, psbH, psbI, psbL, psbT, psbZ*), cytochrome b6f genes (*petA, petB, petD, petG, petL, petN*), ATP synthase genes (*atpA, atpB, atpE, atpF, atpH, atpI*), NADH dehydrogenase genes (*ndhA, ndhB, ndhC, ndhD, ndhE, ndhG, ndhH, ndhI, ndhJ*), transcription genes (*rpoB, rpoC1, rpoC2*), ribosomal protein genes (*rps2, rps11, rps*12, rps14*, rps16, rps18, rpl2, rpl14, rpl16, rpl33*), and other conserved genes such as *cemA, rbcL*, and *pafI*, many of which showed ω values close to zero. Two genes (psaI and rpl33) exhibited neutral evolution (ω = 1), while 13 genes displayed positive selection (ω > 1), including *clp*P, *acc*D, *psb*H, *ccs*A, *cem*A, *ycf*4, *rps*15, *mat*K, *ndh*F, *ycf*2, *ycf*1, *rps*18, and *rps*19. Among these, *ycf*1, rps18, and rps19 showed the highest ω values (2.5,3 and 3, respectively), indicating the strongest signals of positive selection.

### 3.6 Amino acid substitutions

A total of 950 amino acid substitutions were detected across the chloroplast protein-coding genes of *Indigofera*, reflecting substantial protein-level variation (**Supplementary Table 4**). The hypothetical protein gene *ycf1* exhibited the highest number of substitutions (365), followed by *ndh*F (79), *rpo*C2 (64), *ycf*2 (60), and *mat*K (57). Other genes with moderate levels of variation included *acc*D (34), *ccs*A (25), *rpo*B (24), *rpo*A (15), and *ndh*D (14), whereas numerous genes (40 genes) contained only 1–7 substitutions, reflecting strong functional conservation across Indigofera species. Amino acid changes were classified as conservative (94, 9.9%), in which replacements maintained similar physicochemical properties, and radical (856, 90.1%), which involved shifts in charge, polarity, or side-chain size that could impact protein structure and function. The most frequent substitutions included Leucine (L) to Phenylalanine (F, 39), Phenylalanine (F) to Leucine (L, 34), Isoleucine (I) to Leucine (L, 29), Lysine (K) to Asparagine (N, 28), and Isoleucine (I) to Methionine (M, 27), occurring predominantly in highly variable genes.

### 3.7 Phylogenetic analysis

The maximum likelihood (ML) phylogenetic analysis based on complete chloroplast genome sequences, as well as the LSC, SSC, and IR regions, consistently resolved all *Indigofera* species into a strongly supported monophyletic clade, with *Arachis hypogaea* set as the outgroup. The resulting topology was well resolved, exhibiting high bootstrap values (0.98), indicating strong confidence in the inferred relationships. Within *Indigofera*, four distinct clades (A-D) were identified: Clade A (East Asian Clade) comprised *I. carlesii* and *I. hirsuta*; Clade B (Pantropical Clade) included *I. amblyantha* and *I. bungeana*; Clade C (Palaeotropical Clade) contained *I. argentea* and *I. wightii*; and Clade D (Tethyan Clade) grouped *I. linnaei* with *I. linifolia*. All clades received maximal bootstrap support (1.00), confirming clear phylogenetic separation among the species (**Figure 6**).

**Figure 6:**
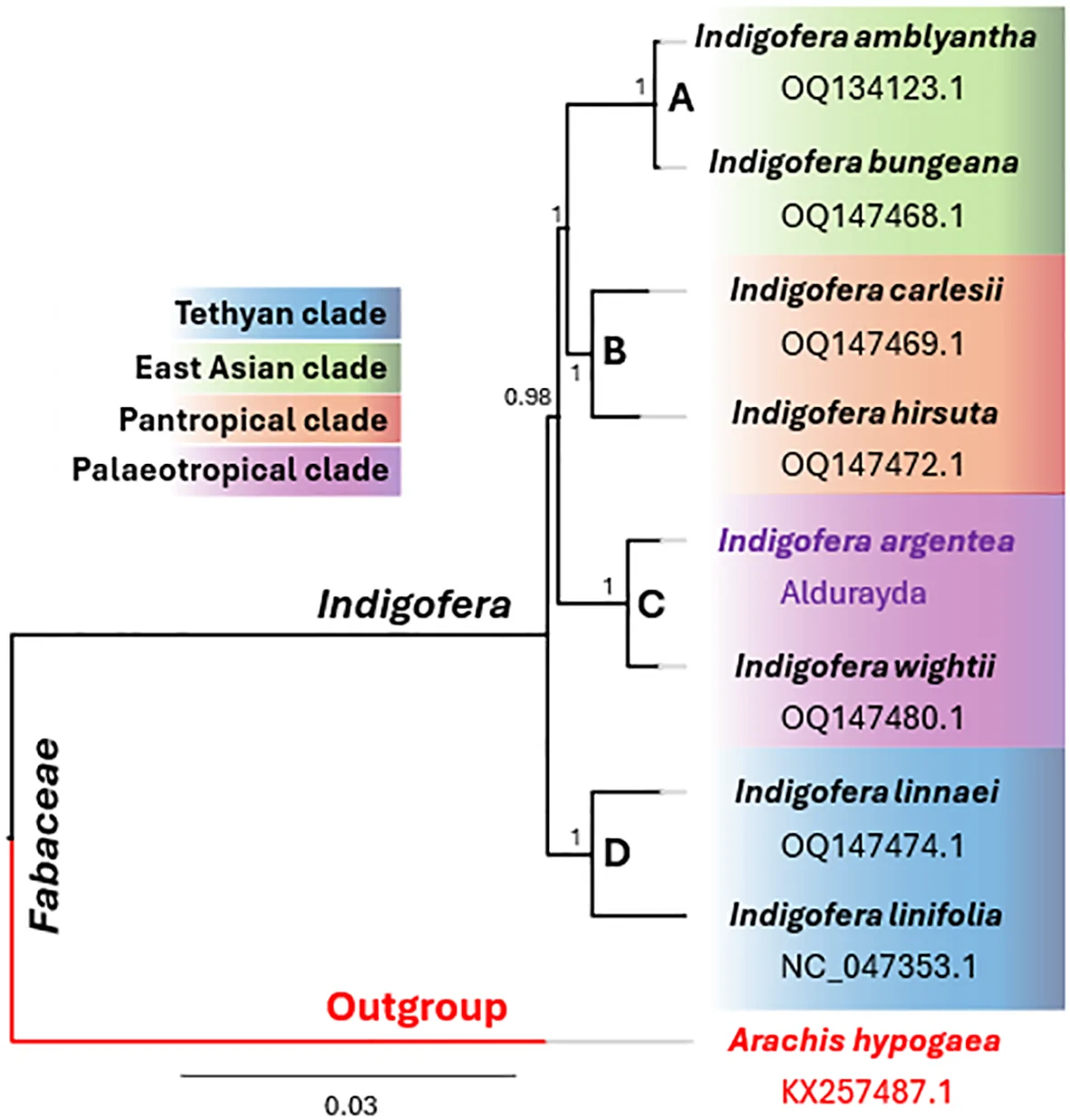
Maximum-likelihood phylogenetic tree constructed from complete chloroplast genome sequences of Indigofera species. *Indigofera argentea* is highlighted in purple. The four major subclades indicated by letters **(A–D)** correspond to the East Asian clade **(A)**, Pantropical clade **(B)**, Palaeotropical clade **(C)**, and Tethyan clade **(D)**, respectively. *Arachis hypogaea* was used as the outgroup to root the tree. Species names are followed by their respective GenBank accession numbers. Bootstrap support values are indicated at the nodes.

## 4 Discussion

Chloroplast genomes have been widely employed in taxonomic and evolutionary studies to elucidate genome structure and resolve phylogenetic relationships among closely related species (Henriquez et al., 2020). In this study, we sequenced and assembled the complete chloroplast genome of *I. argentea* from Saudi Arabia, representing the first reported plastome for this species. The chloroplast genomes of the eight *Indigofera* species analyzed herein exhibited the typical quadripartite structure observed in most angiosperms, comprising the LSC, SSC, and a pair of IR regions. Comparative analysis across *Indigofera* plastomes revealed strong structural conservation, consistent with patterns reported for other legume taxa (Keller et al., 2017; Liao et al., 2021). Genome sizes ranged narrowly from 158,639 bp to 160,040 bp, remaining within the conserved size range characteristic of angiosperm plastomes.

All species encoded an identical set of 128 functional genes, including 83 CDS, 37 trnA genes, and 8 rRNA genes, indicating strong stability in gene composition. Notably, GC content was higher in the IR regions (42.6%) than in the SSC (33.3%) and LSC (27.3%) regions, likely due to the duplication of GC-rich rRNA genes (Lin et al., 2025), a characteristic feature of most angiosperm plastomes (Kiktev et al., 2018). This conserved gene complement aligns with the findings of Zhou et al (Zhou et al., 2023), supporting the structural stability of *Indigofera* plastomes. The maintenance of essential chloroplast genes suggests strong functional constraints during plastome evolution (Wicke et al., 2011), whereas species differentiation may be reflected primarily by localized sequence variation rather than major changes in genome structure (Zhou et al., 2023). The addition of *I. argentea* further expands available genomic resources and enhances our understanding of plastome conservation and evolution within the genus.

A codon is a triplet of nucleotides encoding a specific amino acid, with 64 codons specifying 20 amino acids (Crick, 1962). Synonymous codons encode the same amino acid but may be used at different frequencies due to mutational or selective biases (Sharp and Matassi, 1994), with factors influencing codon usage including natural selection and genetic mutation (Parvathy et al., 2022). The RSCU measures this bias, where RSCU = 1 indicates no preference, >1 indicates preferred codons, and <1 indicates underrepresented codons (Sharp and Li, 1986). In the *I. argentea* chloroplast genome, 25,611 codons were identified, with methionine (AUG) and tryptophan (UGG), with no codon preference detected, consistent with the results of Luo et al. (2021), while leucine, serine, and arginine, each encoded by six codons, exhibited the highest degeneracy. Most amino acids preferred A- or U-ending codons, and high-frequency codons largely ended in A/U, reflecting the AT-rich composition of the plastome and supporting their role in molecular evolution (Sharp and Li, 1986; Campbell and Gowri, 1990). This pattern is consistent with previously reported Indigofera plastomes, suggesting a conserved codon usage bias across the genus rather than substantial lineage-specific shifts (Zhao et al., 2023; Zhou et al., 2023). The preference for A or U at the third codon position may be influenced by mutation pressure and selective constraints, as synonymous substitutions at this position can be relatively neutral (Zhang et al., 2007). However, the observed nucleotide bias suggests that codon usage is shaped not only by mutation pressure but also by natural selection, as reported in other plant lineages, including Asteraceae (Nie et al., 2014) and Euphorbiaceae (Wang et al., 2020).

Repeat sequences, abundant and widely dispersed in chloroplast genomes, play key roles in genome organization and evolutionary dynamics and are widely used as informative markers in population genetics, phylogenetics, and evolutionary studies (Liang et al., 2019; Vining et al., 2019). The *I. argentea* chloroplast genome contains diverse SSRs, mostly dinucleotide and trinucleotide repeats, predominantly in the LSC region, highlighting its role as a genetic reservoir (Vining et al., 2019). Comparative analysis across eight *Indigofera* plastomes revealed conserved SSR distributions, with AT-rich repeats dominating, reflecting typical angiosperm patterns (Kong and Yang, 2017; Huang et al., 2022). The conserved yet species-specific SSR profiles observed here provide valuable resources for developing molecular markers in *Indigofera*. The conserved yet species-specific SSR profiles identified here provide useful resources for assessing genetic diversity, characterizing germplasm, tracing maternal lineages, and supporting the conservation and management of Indigofera genetic resources (Khade et al., 2025).

The contraction and expansion of IR regions are important sources of plastome size variation (Goulding et al., 1996; Knox and Palmer, 1999). Analysis of IR/SC junctions provides valuable insight into interspecific variation driven by these changes. Comparative analysis revealed highly conserved IR boundaries across the eight *Indigofera* plastomes, with only minor shifts in *rps*19, *ycf*1, and *ndh*F at the junctions, consistent with patterns reported in other angiosperms (Du et al., 2022; Zhou et al., 2023). These conserved junctions, comparable to those observed in related Fabaceae plastomes, indicate limited IR expansion and contraction within Indigofera and overall stability of plastome organization (Zhao et al., 2023; Zhou et al., 2023). The observed boundary variations may nevertheless represent lineage-specific structural changes and provide potentially useful markers for species identification, while also contributing to pseudogene formation (Guisinger et al., 2011; Wicke et al., 2011).

Nucleotide diversity analysis demonstrated that sequence variation in *Indigofera* plastomes is concentrated within non-coding regions, a pattern consistently observed across angiosperm chloroplast genomes. In line with this, sliding-window analysis identified four mutation hotspot regions, trnK-*rbc*L, trnL-*rps*4, *ndh*F-trnL, and *ycf*1, which exhibited elevated nucleotide diversity and represented promising candidates for interspecific genetic differentiation and molecular marker development (Wang et al., 2023a; Liang et al., 2024). Among coding regions*, ycf*1 showed particularly high variability, reinforcing its established utility as a highly informative plastid barcode and a phylogenetic markers for species delimitation within the genus (Shaw et al., 2005).

The distribution of SNPs across *Indigofera* chloroplast coding regions reflects typical patterns of plastome evolution, with uneven mutation rates among genes and codon positions. Transition and transversion substitutions were unevenly distributed, with a predominance of transitions and a majority occurring at the third codon position, indicating strong functional constraints and selective neutrality at synonymous sites (Cox et al., 2014). At the gene level, highly polymorphic loci such as *ycf*1, *rpo*C2, and *ndh*F exhibited the greatest sequence variation, whereas several genes involved in photosynthesis and ribosomal function were highly conserved, underscoring their essential roles in chloroplast function. These patterns highlight that, while the plastome is overall conserved, localized regions of elevated variation may contribute to adaptive divergence and provide informative markers for phylogenetic and evolutionary studies (Cauz-Santos, 2025). However, the present data support these regions primarily as indicators of evolutionary divergence and potential adaptive differentiation, rather than providing direct evidence of adaptation to specific environmental conditions.

Positive selection drives adaptive evolution, while purifying selection predominates to maintain essential chloroplast functions (Moseley et al., 2018). In *Indigofera*, the majority of coding sequences (59 genes) exhibited low dN/dS ratios, indicating strong purifying selection, while a smaller subset of 13 genes showed signatures of positive selection at specific codon sites. This pattern is consistent with previous Indigofera plastome studies, which reported predominant purifying selection and positive selection in a smaller subset of genes (Zhou et al., 2023). Notably, several genes identified here (*acc*D, *ccs*A, *mat*K, and *ndh*F) have also been reported to be under positive selection in previous Indigofera studies, suggesting their potential importance in lineage-specific evolution despite the overall conservation of chloroplast genomes. These genes are likely associated with critical chloroplast functions, including photosynthesis, stress response, and leaf development; and the observed positive selection signals may reflect historical evolutionary pressures, although their association with specific environmental factors such as heat and drought remains to be experimentally validated (Martín et al., 1996; Madoka et al., 2002).

Evolution drives changes in a protein’s sequence over time (Doud et al., 2015), raising the question of how these substitutions affect structure and function. In *Indigofera* chloroplasts, 950 amino acid substitutions were detected, with 90.1% radical and 9.9% conservative, indicating potential changes in protein structure and interactions (Miyata et al., 1979). Highest variability occurred in *ycf1*, followed by ndhF, *rpo*C2, *ycf*2, and *mat*K, identifying these genes as mutation hotspots within the plastome. These non-random substitutions reflect localized evolutionary pressures and highlight targets for phylogenetic studies, molecular marker development, and future investigations of adaptive evolution. The concentration of amino acid substitutions in a limited number of genes, alongside the strong conservation of many other plastid proteins, further supports a heterogeneous pattern of molecular evolution within the *Indigofera* plastome. Such localized divergence may contribute to lineage-specific differentiation while preserving the core functions required for chloroplast activity (Zhou et al., 2023).

Although *Indigofera* comprises more than 750 species, the present taxon sampling was guided by the established phylogenetic framework of the genus, with emphasis on species from the same or closely related clades to *I. argentea* (Schrire, 2013; Zhou et al., 2023). This targeted strategy was adopted to assess the phylogenetic placement of I. argentea within its closest evolutionary context. The chloroplast phylogenetic trees were used to assess the taxonomic and evolutionary relationships among the plant species (Wang et al., 2023b). The ML phylogeny based on complete plastome sequences recovered *Indigofera* as a strongly supported monophyletic lineage (BS = 1; **Figure 6**), consistent with previous plastid phylogenomic studies (Zhou et al., 2023) that recognized Pantropical, East Asian, Tethyan, and Palaeotropical lineages. In our topology, Clade A (*I. carlesii* and *I. hirsuta*) corresponds to the Pantropical lineage, Clade B (*I. amblyantha* and *I. bungeana*) to the East Asian lineage, and Clade C (*I. argentea* and *I. wightii*) to the Palaeotropical lineage, whereas Clade D (*I. linnaei* and *I. linifolia*) represents the Tethyan lineage, supporting earlier biogeographic classifications of the genus proposed by Zhou et al (Zhou et al., 2023). Notably, in their phylogenomic study of 18 Indigofera chloroplast genomes, Zhou et al (Zhou et al., 2023). included only a single representative of the Palaeotropical clade (*I. wightii*), limiting insights into its internal diversification. By including *I. argentea*, which clusters within this lineage based on phylogenetic placement and biogeographic affinities, our study provides a more complete representation of this lineage. These results underscore the need for broader taxon sampling in future phylogenomic analyses to resolve the monophyly, internal structure, and evolutionary history of the Palaeotropical clade.

Integrated analyses of nucleotide diversity, SNPs, positive selection, and amino acid substitutions highlighted several plastome regions as highly promising molecular markers for *Indigofera*. Among coding genes, *ycf*1 exhibited the highest variability, reinforcing its recognition as an exceptionally informative plastid barcode across angiosperms (Dong et al., 2015; Li et al., 2021; Jiang et al., 2022; Zhou et al., 2023), facilitating clear differentiation among closely related taxa. Other coding genes, including *ndh*F*, rpo*C2, and *ycf*2, also showed elevated variability; however, their potential as molecular markers remains underexplored and warrants further validation (Huang et al., 2010; Li et al., 2021). In addition, several intergenic spacers, such as trnK-*rbc*L, trnL-*rps*4, and *ndh*F-trnL, demonstrated high variability, with *trn*K-*rbc*L and *ndh*F-trnL previously reported as informative in *Indigofera* (Zhou et al., 2023). Collectively, these coding and non-coding regions represent promising candidate markers for species identification, phylogenetic reconstruction, and evolutionary studies in *Indigofera*, although their utility should be further validated across broader taxon sampling and independent datasets.

## 5 Conclusion

The complete chloroplast genome of *Indigofera argentea* from Saudi Arabia was assembled and compared with seven related *Indigofera* species, providing a valuable plastome resource for this species. While the genome exhibits the typical quadripartite structure with conserved gene content, gene order, and IR boundaries, variations in SSR distribution, codon usage bias, and highly variable coding and non-coding regions reveal informative signals for evolutionary studies and DNA barcoding. Selection analyses revealed predominantly purifying selection, with a subset of genes under positive selection, suggesting potential adaptive evolution. Plastome-based phylogeny robustly supported the monophyly of *Indigofera* and provided clear resolution of evolutionary relationships among its lineages. Overall, this study provides a comprehensive genomic framework for taxonomic resolution, conservation planning, and future evolutionary and population genetic studies of *Indigofera* species in Saudi Arabia and beyond. Integration of complementary nuclear genomic data in future studies may further enhance the understanding of evolutionary relationships and diversification within the genus.

## Data Availability

The complete chloroplast genome sequence of *Indigofera argentea* is publicly available in the NCBI GenBank database under accession number PZ945921.
